# Supplementary material for: Complexome profiling of the Chlamydomonas psb28 mutant reveals TEF5 as an early PSII assembly factor
Source: Plant Cell. 2025 Mar 17;37(6):koaf055. doi: 10.1093/plcell/koaf055 (PMC12164590; doi:10.1093/plcell/koaf055)
Supplement: koaf055_Supplementary_Data [file koaf055_supplementary_data.zip › psb28_interactive.zip!/plots/intensity/qualityPlots/peptides/TGD1_Cre06.g268200.t1.2.html]

### Cre06.g268200.t1.2

Trivial name: TGD1  
SeqCoverage [median]: 15.20  
Mass: 36.47 kDa  
  
 

| Sequence | MissedCleavage | Mass [Da] | wtIntensity | muIntensity | Score |
| --- | --- | --- | --- | --- | --- |
| ELTPVVTAIILAGR | 0 | 1451.87 | 528.28 | 0.00 | 55.40 |
| NTAEQLR | 0 | 830.42 | 0.00 | 946.20 | 99.14 |
| SVGGVLALALSR | 0 | 1141.68 | 359.35 | 0.00 | 78.65 |
|  |  |  | 887.6 | 946.2 |  |
